# Myostatin Mutation in Japanese Quail Increased Egg Size but Reduced Eggshell Thickness and Strength

**DOI:** 10.3390/ani12010047

**Published:** 2021-12-27

**Authors:** Joonbum Lee, Cameron McCurdy, Christopher Chae, Jinwoo Hwang, Madeline C. Karolak, Dong-Hwan Kim, Cassandra L. Baird, Benjamin M. Bohrer, Kichoon Lee

**Affiliations:** 1 Department of Animal Sciences, The Ohio State University, Columbus, OH 43210, USA; lee.3920@osu.edu (J.L.); mccurdy.114@osu.edu (C.M.); karolak.6@osu.edu (M.C.K.); kim.4094@osu.edu (D.-H.K.); cassandra.l.baird@wilmington.edu (C.L.B.); bohrer.13@osu.edu (B.M.B.); 2 The Ohio State University Interdisciplinary Human Nutrition Program, The Ohio State University, Columbus, OH 43210, USA; 3 Department of Materials Science and Engineering, The Ohio State University, Columbus, OH 43212, USA; chae.85@buckeyemail.osu.edu (C.C.); hwang.458@osu.edu (J.H.)

**Keywords:** myostatin, quail, eggshell, scanning electron microscopy

## Abstract

**Simple Summary:**

Avian eggs provide huge benefits to both science and society by providing an important model for developmental studies and a high-quality protein source for the human diet. Especially, the hard-shell layer existing at the outer part of eggs is a unique characteristic, which is exclusive in avian species compared to other egg-laying species. Among various avian models developed to investigate genetic factors for potential industrial application, myostatin (MSTN) mutations in quail and chickens were recently generated, resulting in improved meat yield. In addition to previously reported growth and egg production traits in MSTN mutant quail, eggshell quality of mutants was further investigated in this study. Although eggshell height, width, and weight were increased by the MSTN mutation, eggshell breaking strength (EBS) and eggshell thickness were decreased in mutant eggs compared to wild-type eggs. Although these data indicated that decreased eggshell thickness contributed to decreased EBS in mutant eggs, the cellular mechanism of thinner eggshell formation in uterus by MSTN mutation needs to be further investigated using MSTN mutant quail.

**Abstract:**

Recently developed myostatin (MSTN) mutant quail and chickens demonstrated similar effects of MSTN on muscle and fat developments between avian and mammalian species. However, the effect of MSTN mutation on the quality of eggshells, an important avian specific characteristic, has not yet been investigated although egg production traits of mutant quail have been studied. In this study, several parameters for eggshell quality, including eggshell size, eggshell weight, eggshell breaking strength (EBS), and eggshell thickness, were all compared between MSTN mutant and wild-type (WT) eggs. MSTN mutant eggs had greater height and width along with heavier eggshell weight compared to WT eggs, which shows proportional improvement in egg size as affected by the MSTN mutation. However, EBS and eggshell thickness were decreased in mutant eggs compared to WT eggs. In addition, the palisade layer, the thickest and most important layer for the strength of an eggshell, was also decreased without a change in the number of vesicular holes. These data indicated that decreases in the thickness of the eggshell and the palisade layer would be a main factor contributing to a lower EBS in mutant eggs. MSTN mutant quail provide a useful model to better understand the function of MSTN on avian uterine cell development and eggshell biomineralization.

## 1. Introduction

The anti-myogenic function of myostatin (MSTN) was discovered from the experiment generating MSTN knockout mice [1] and further demonstrated in other MSTN mutant animals and humans [2,3,4,5,6,7,8,9]. Due to the double-muscled phenotype in major livestock animals carrying MSTN mutation [2,3,4,5,6], industrial applications of the MSTN mutations as a potential selection marker for improved growth traits and increased meat yield has been an ongoing research topics in animal science. Since the generation and identification of MSTN mutant mice and cattle [1,2,3], respectively, various mammalian models carrying the MSTN mutation have been developed and identified to investigate MSTN functions in mammals for more than 20 years. By comparison, avian models with MSTN mutation were more recently generated [10,11]. Due to MSTN mutant chickens and quail having higher muscle mass and lower fat deposition, the major functions of MSTN on muscle and adipose tissues are conceptually similar between mammalian and avian species. However, additional research regarding MSTN functions on avian-specific characteristics is still needed.

Among avian-specific characteristics, egg production is an important characteristic in birds that offers huge benefit on developmental studies in science and provides a high-quality protein source for the human diet. Although there are many species of animals that lay eggs, including fish, reptiles, amphibians, and insects, avian eggs are unique, as a hard-shell layer exists as an outer part of the egg to protect inside contents from external impacts. On the surface of an uncalcified eggshell membrane, calcified and mineralized shell layers are formed in the order of the mammillary layer, the palisade layer, the vertical crystal layer, and the outermost cuticle layer [12]. There are several factors affecting eggshell strength across different avian species, such as the egg weight and the crystallographic orientation of the mineralized eggshell [13,14]. Because the same species have the same crystallographic orientation, however, eggshell thickness is a main factor affecting eggshell strength [15]. Most notably, thickness of the palisade layer, a major calcified layer in the eggshell [12], is more correlated to the eggshell strength than whole eggshell thickness [16].

In our previous study, MSTN mutant quail were generated using the adenoviral CRISPR/Cas9 system [10]. Active research investigating the phenotypic characteristics of MSTN mutant quail revealed the positive effects of MSTN mutation on growth, muscle mass, and feed efficiency along with reducing fat deposition [17], collectively suggesting MSTN as a potential selection maker to improve the desirable traits in the broiler industry. Furthermore, evaluation of egg-production traits of MSTN mutant quail provided meaningful information that may also be considered in the layer industry [18]. Despite multiple research efforts using MSTN mutant birds, changes in eggshell quality by MSTN mutation have not yet been investigated. Therefore, the objective of this study was to investigate the eggshell quality of MSTN mutant eggs by examination of egg height, width, weight, and eggshell breaking strengths (EBS). In addition, scanning electron microscopy was further used to analyze the thickness of the entire eggshell and the palisade layer.

## 2. Materials and Methods

### 2.1. Animal Care

Japanese quail (*Coturnix japonica*) with MSTN mutation were obtained from our previous study [10] and maintained in The Ohio State University (OSU) Poultry Facility in Columbus, Ohio. All animals were fed ad libitum and raised together in a battery cage until the egg-collection period for the experiment. After the completion of egg collection, quail used in this study were euthanized via CO_2_ inhalation. All experimental procedures and animal care protocols were approved by the Institutional Animal Care and Use Committee of OSU (Protocol 2019A00000024, Approval date: 11 March 2019).

### 2.2. Collection and Analysis of Eggs and Eggshells

At 4 months of age, the female quail were transferred into individual cages. After adaptation periods, eggs were collected from 10 MSTN mutant females and 10 wild-type (WT) females daily, and they were stored in an egg cooler maintained at 15 °C with 80% relative humidity for the experiment conducted every five days. From each female, a total of 10 eggs were used to measure egg height, egg width, egg weight, eggshell weight, and EBS. Egg height and egg width were measured using a digital caliper (±0.01 mm), and egg weights were measured using a laboratory balance. To estimate the eggshell curvature, height to width ratio was also calculated. Subsequent to such initial measurements, EBS was measured using a material testing machine equipped with a TA-25A acrylic cylinder probe (5.08-cm diameter, 20-mm tall) (Stable Micro Systems TA. XT plus 100C, Stable Micro System Corp, Surrey, UK). To measure the EBS, eggs were positioned vertically by placing the blunt-end “up” and pointed-end “down”. The eggs were broken with a pre-test and test speed of 2.0 mm/s and a set distance of 2.0 mm. The peak force during the test was reported. After the EBS measurement, the blunt end of the eggs was slightly crushed, and these eggs were neatly cut in half through the equator to remove egg whites and yolks. Subsequently, the eggshells were washed with water, dried at room temperature overnight, and then weighed the next day. Any pieces of eggshell that came off during the processes were collected and weighed together with the entire eggshell.

### 2.3. Sample Preparation for Scanning Electron Microscopy

After the measurement of the eggshell weights, two eggshells were randomly selected from each of five MSTN mutant and WT females and prepared for ultrastructural examination by cutting into a small piece (1 cm^2^) of the eggshell taken from the equatorial region. One specimen from each egg was mounted vertically using carbon paint on aluminum stubs. Subsequently, Thermo Scientific Apreo FEG scanning electron microscope (Thermo Fisher Scientific Inc., Waltham, MA, USA) was used to acquire micrographs of samples at an accelerating voltage of 10.00 kV and current of 0.10 nA. The microscope was operated in its “low vacuum” mode, where water vapor was introduced into the chamber to partially neutralize the electron beam since the samples are non-conducting. To measure the eggshell thickness and the number of vesicular holes within the palisade layer, one micrograph showing cross-section of the entire eggshell and five randomly taken micrographs showing the cross-section of the palisade layer were obtained from each egg at a magnification of × 650 and × 10,000, respectively. The thicknesses of the entire eggshell and the palisade layer and the number of vesicular holes in a square area of 5 μm × 5 μm within each micrograph of the palisade layer were measured using NIH image J software (ImageJ, Ver. 1.52, http://imagej.nih.gov/ij).

### 2.4. Statistical Analyses

Student’s *t*-test was used for statistical analysis to compare all measurements between the MSTN mutant eggs and the WT eggs. All data were expressed as means ± standard error of the mean and performed by the GraphPad PRISM (ver. 6.02)

## 3. Results

### 3.1. The Effects of MSTN Mutation on Eggshell Size and Breaking Strength

The average sizes of the MSTN mutant eggs were larger, since the height and width of the mutant eggs were significantly increased by approximately 2% and 3%, respectively, compared to those of the WT eggs. (Table 1). In addition to increased size, the weights of the eggshells and the entire eggs were significantly increased by approximately 5.5% and 8.5%, respectively, when the mutant group was compared to the WT group. However, EBS was significantly decreased in the MSTN mutant eggs compared to the WT eggs, indicating a weaker eggshell in the MSTN group.

### 3.2. The Effects of MSTN Mutation on Structural Properties of Eggshell and Palisade Layer

**Figure 1:**
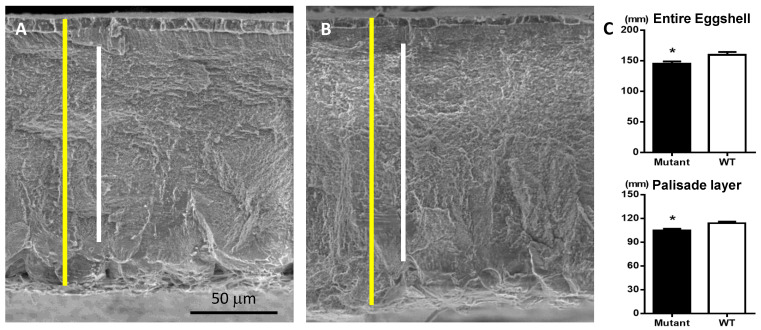
Cross-section of entire eggshell. (**A**) Scanning electron microscopy (SEM) image of MSTN mutant eggshell. (**B**) SEM image of WT eggshell. (**C**) Comparisons of the length of entire eggshell and palisade layer. White bars represent the thickness of eggshell from cuticle layer to mammillary layer; yellow bars represent the thickness of palisade layer; * *p* < 0.05.

Whole eggshell thickness was measured from the top of the cuticle layer to the bottom of the mamillary layer, and the palisade layer referred to the layer between the vertical crystal layer and the mammillary layer (Figure 1A,B). The length of the eggshell thickness in the mutant eggs was significantly shorter than those in the WT eggs (145.43 μm vs. 159.74 μm) (Figure 1C). Likewise, the length of the palisade layer thickness was significantly shorter in mutant eggs compared to WT eggs (104.66 μm vs. 113.89 μm) (Figure 1C).

**Figure 2:**
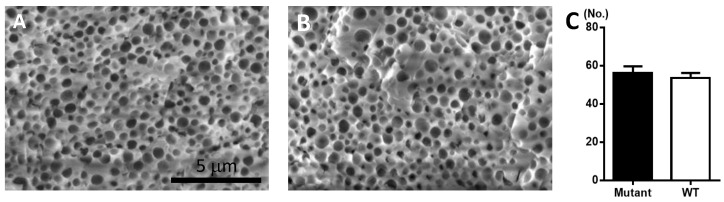
Cross-section of palisade layer in quail egg. (**A**) SEM image of the palisade layer in MSTN mutant egg. (**B**) SEM image of the palisade layer in WT egg. (**C**) Comparison of the number (No.) of vesicular holes in a square area of 5 μm × 5 μm within the palisade layer.

In addition to the thickness, the number of vesicular holes within the palisade layer, another structural property of the eggshells, were compared between mutant and WT eggs (Figure 2A,B). The average number of vesicular holes within the unit area of the palisade layer was not significantly different between these two groups (Figure 2C).

## 4. Discussion

The previous study, focusing on reproductive traits of MSTN mutant females, reported that, from 90 days to 110 days of age, MSTN females lay significantly heavier eggs compared to WT females [18]. The heavier eggs from MSTN mutant females were continuously observed after four months of age in this study (Table 1). In addition, increased eggshell weight contributed to the growth in egg weight in MSTN mutant quail. Significant increases in both height and width of eggs increased the volume of mutant eggs, further supporting the increased weights of MSTN mutant eggs (Table 1).

Along with eggshell size and weight, EBS is another important trait to be considered in egg quality. In the current study, EBS was significantly reduced in the mutant eggs compared to the WT eggs (Table 1). To explain such results, eggshell thickness and curvature were further analyzed because eggshell thickness is positively correlated with eggshell strength within the same species and large curvature of eggshell without a change in thickness can decrease eggshell strength [15,19]. There were similar ratios of height to width between groups (Table 1), suggesting similar curvatures between two groups. However, eggshell thickness was significantly decreased in mutant eggs compared to WT eggs (Figure 1C). These data indicated that a thinner eggshell caused by MSTN mutation would be one of the main factors affecting eggshell strength in this study. A thin eggshell of mutant eggs might have a negative effect on the protective role of an eggshell from external physical impacts and bacterial penetration [20]. Moreover, it has been reported that a thinner eggshell is associated with poor hatchability in both chickens and quail [21]. However, the number of cracked/broken eggs were not noticeable in both groups during consistent egg collection, and the hatchability of mutant eggs was almost the same with that of WT eggs in our previous study [18], indicating these reduction rates of the eggshell parameters caused by the MSTN mutation may not be practically significant.

Among four different layers within an eggshell, the palisade layer is the thickest and most related with eggshell strength due to its thickness and calcified properties [16]. Therefore, decreased thickness of the palisade layer, in addition to whole eggshell thickness, contributed to the lower EBS in mutant eggs compared to WT eggs (Figure 1C). However, similar proportions of the palisade layer, approximately 70% in both groups, without significant changes in the number of vesicular holes within the palisade layer between mutant and WT eggs (Figure 2C) indicated that the effect of MSTN on an eggshell might be more general throughout eggshell layers than they are specific to the palisade layer. The effect of MSTN mutation on the palisade layer thickness without a difference on the number of vesicular holes again confirmed the association between palisade layer thickness and eggshell strength in this study.

Although MSTN is known to be expressed mainly in muscle, expression of MSTN in other tissues and physiological functions of MSTN in these tissues have been reported. In utero, when an eggshell is formed, the relative expression level of MSTN was significantly lower in laying hens compared to non-laying hens of the same age [22], indicating regulation of MSTN expression during eggshell biomineralization. MSTN mutant quail provide a valuable model for future research initiatives focused on developing a further understanding for the function of MSTN on avian uterine cells and their regulation of eggshell biomineralization.

## 5. Conclusions

Other than major functions of MSTN on muscle and fat tissues, the function of MSTN on eggshell qualities was investigated by using MSTN mutant quail for the first time. Although MSTN mutant eggs showed increased height, width, and weight of eggshells, the thickness was reduced in mutant eggs compared to WT eggs. Notably, eggshell breaking strength was lower in mutant eggs compared to that of WT eggs, which is associated with thinner eggshells and thinner palisade layers. Further studies using MSTN mutant quail will provide a more detailed understanding for the mechanism of MSTN on regulation of avian uterine development and eggshell biomineralization.

## Figures and Tables

**Table 1:** Comparisons of eggshell qualities between myostatin (MSTN) mutant eggs and wild-type (WT) eggs.

	Mutant	WT	*p*-Value
Height (mm)	31.45 ± 0.16	30.79 ± 0.20	0.0208
Length (mm)	24.65 ± 0.10	23.95 ± 0.10	0.0001
Height/Length	1.29 ± 0.02	1.29 ± 0.01	NS
Egg Weight (g)	10.33 ± 0.12	9.5 ± 0.11	0.0001
Eggshell Weight (g)	0.74 ± 0.01	0.71 ± 0.01	0.0347
Eggshell Breaking Strength (kg)	1.24 ± 0.04	1.4 ± 0.06	0.0276

The values are mean ± standard error of the mean. Student *t*-test was used for statistical analysis. NS, not significant; *p* < 0.05.
